# Supplementary material for: Inhibition of ADAM17 increases the cytotoxic effect of cisplatin in cervical spheroids and organoids
Source: Front Oncol. 2024 Sep 2;14:1432239. doi: 10.3389/fonc.2024.1432239 (PMC11402614; doi:10.3389/fonc.2024.1432239)
Supplement: Supplementary file 1 [file DataSheet1.docx]

Data Sheet 1

Supplementary Material

Inhibition of ADAM17 increases cytotoxic effect of cisplatin in cervical spheroids and organoids

**David Holthaus ^1,2 †^, Christoph Rogmans ^1 †^, Ina Gursinski ^1^, Alvaro Quevedo-Olmos ^2^, Marzieh Ehsani ^2^, Mandy Mangler ^3,4^, Inken Flörkemeier ^1^, Jörg P. Weimer ^1^, Thomas F. Meyer ^2^, Nicolai Maass ^1^, Dirk O. Bauerschlag ^1,5^, Nina Hedemann ^1 *^**

^1^ Department of Gynaecology and Obstetrics, University Hospital Schleswig-Holstein, Kiel, Germany

^2^ Laboratory of Infection Oncology, Institute of Clinical Molecular Biology, Christian-Albrechts-Universität zu Kiel and University Hospital Schleswig-Holstein, Kiel, Germany

^3^ Department of Gynaecology and Obstetrics, Vivantes Auguste Viktoria-Klinikum, Berlin, Germany

^4^ Department of Gynaecology, Charité University Medicine, Berlin, Germany

^5^ Clinic and Polyclinic for Gynaecology and Reproductive Medicine, University Hospital Jena, Jena, Germany

^†^ These authors contributed equally to this work and share first authorship

*** Correspondence:**Dr. Nina Hedemann

Email: nina.hedemann@uksh.de

# Supplementary methods

**Triple staining of spheroid cultures**

To determine cell numbers for spheroid generation, 100-10,000 cells were seeded in ultra-low attachment, black-transparent 96-well plates (Corning, 4520). Cells were grown for 24 (CaSki) or 96 (SIHA and C33A) hours. Then, 5 µg/ml Hoechst33342 (Thermo Fisher, H1399), 0.2 µg/ml Propidium iodide (Miltenyi Biotec, 130-093-233), and 1 ng/ml Calcein-AM (Invitrogen, C34852) was added to the cells. Following a three-hour incubation at room temperature, cells were imaged with the NyOne imaging system (SYNENTEC). Images were exported using YT software (SYNENTEC) and scale bars were added in FIJI.

# Supplementary Figures


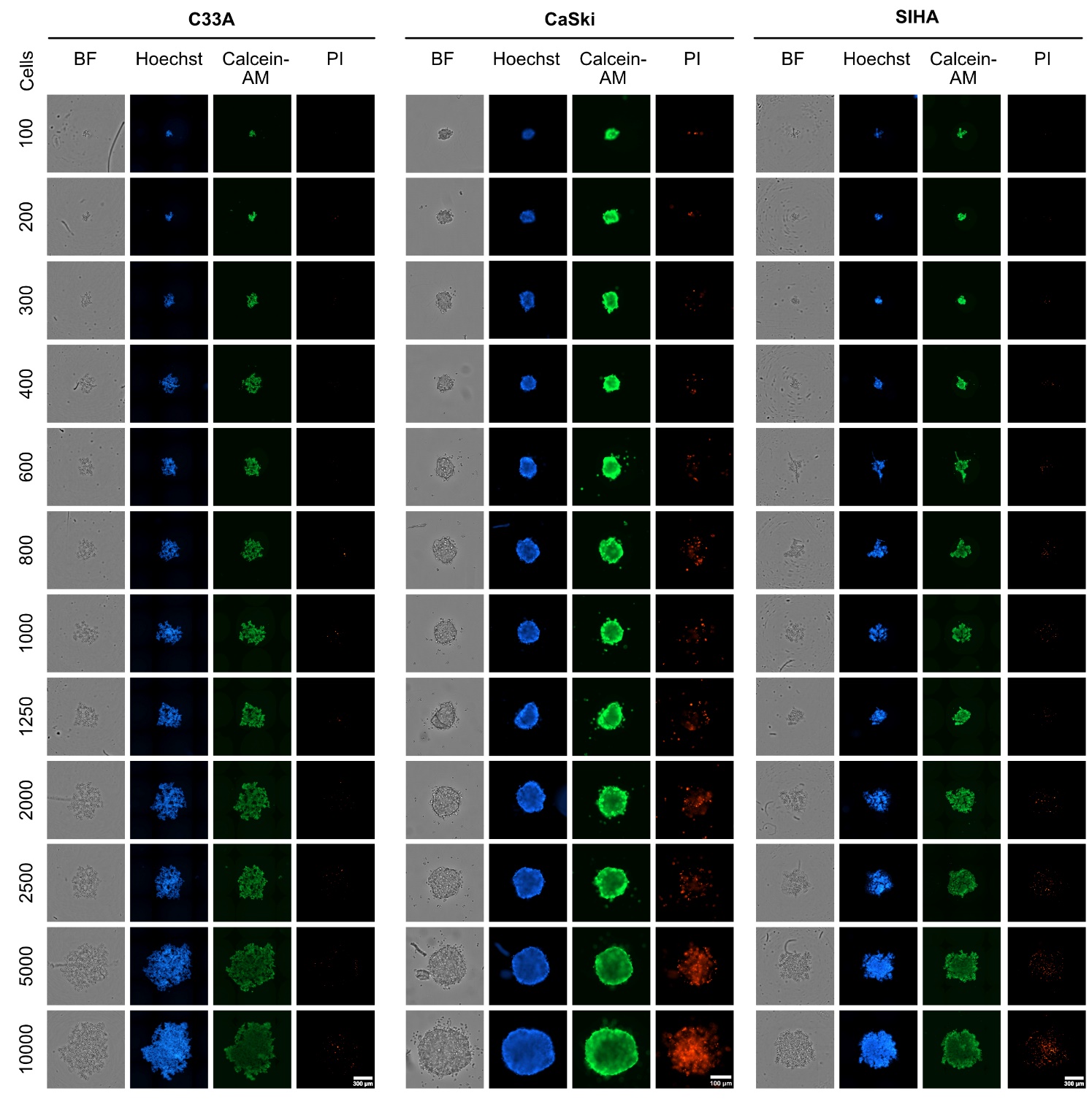


Supplementary Figure 1: Triple staining of spheroids composed of various cell numbers of C33A, CaSki and SIHA cells. Cells were stained with Hoechst33342 (blue), Calcein-AM (green) and propidium iodide (PI, red). For later experiments, 10,000 C33A cells, 7,000 CaSki cells and 5,000 SIHA cells were used as necrotic core formation as observed by PI staining was low. Scale bars indicate 300 µm for C33A and SIHA, and 100 µm for CaSki.


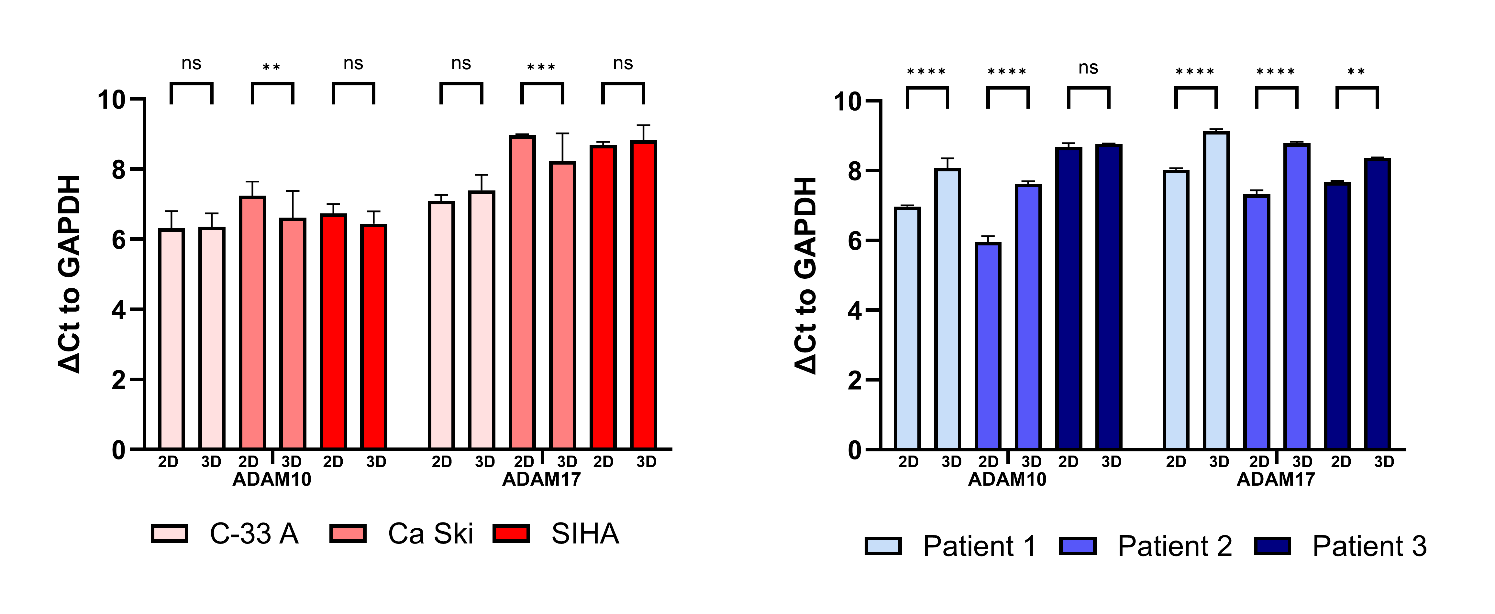


Supplementary Figure 2: Transcriptional expression of ADAM10/17 normalized to GAPDH as measured by RT-qPCR. Data shows mean (± SEM) from 3 independent experiments. Statistical significance was determined using a Two-Way ANOVA with Tukey’s correction for multiple testing. ns not significant * p < 0.05, ** p < 0.01, *** p < 0.001**** p < 0.0001.


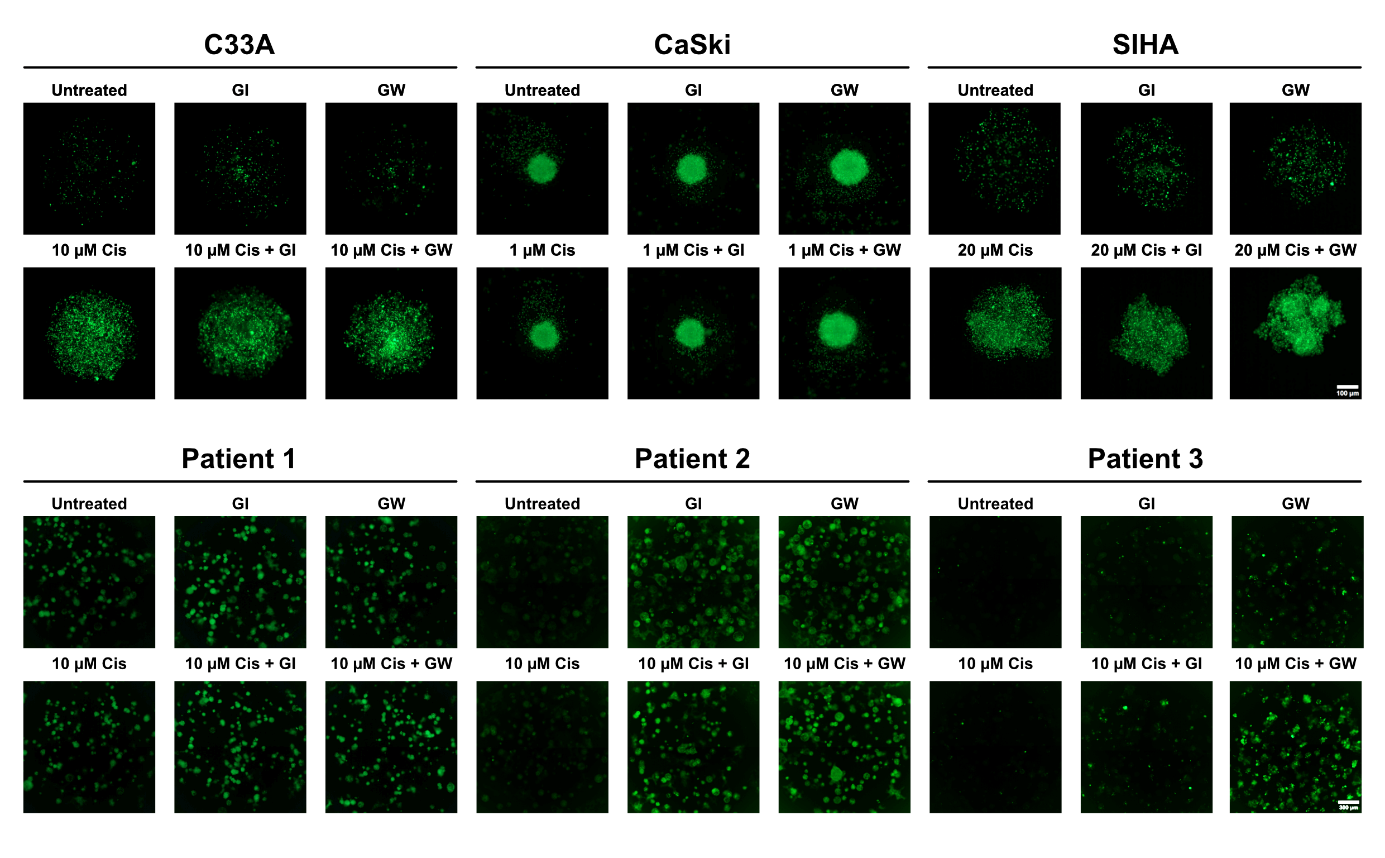


Supplementary Figure 3: Representative images of CellTox Green staining after 36 (cell lines) or 72 (organoids) hours of incubation. Spheroids or organoids were either untreated, treated with ADAM10 inhibitor GI254023X, treated with ADAM10/17 inhibitor GW280264X, or treated with combinations of cisplatin and ADAM inhibitors. CellTox green marks dead cells. Scale bars indicate 100 µm for cell lines and 300 µm for organoids.


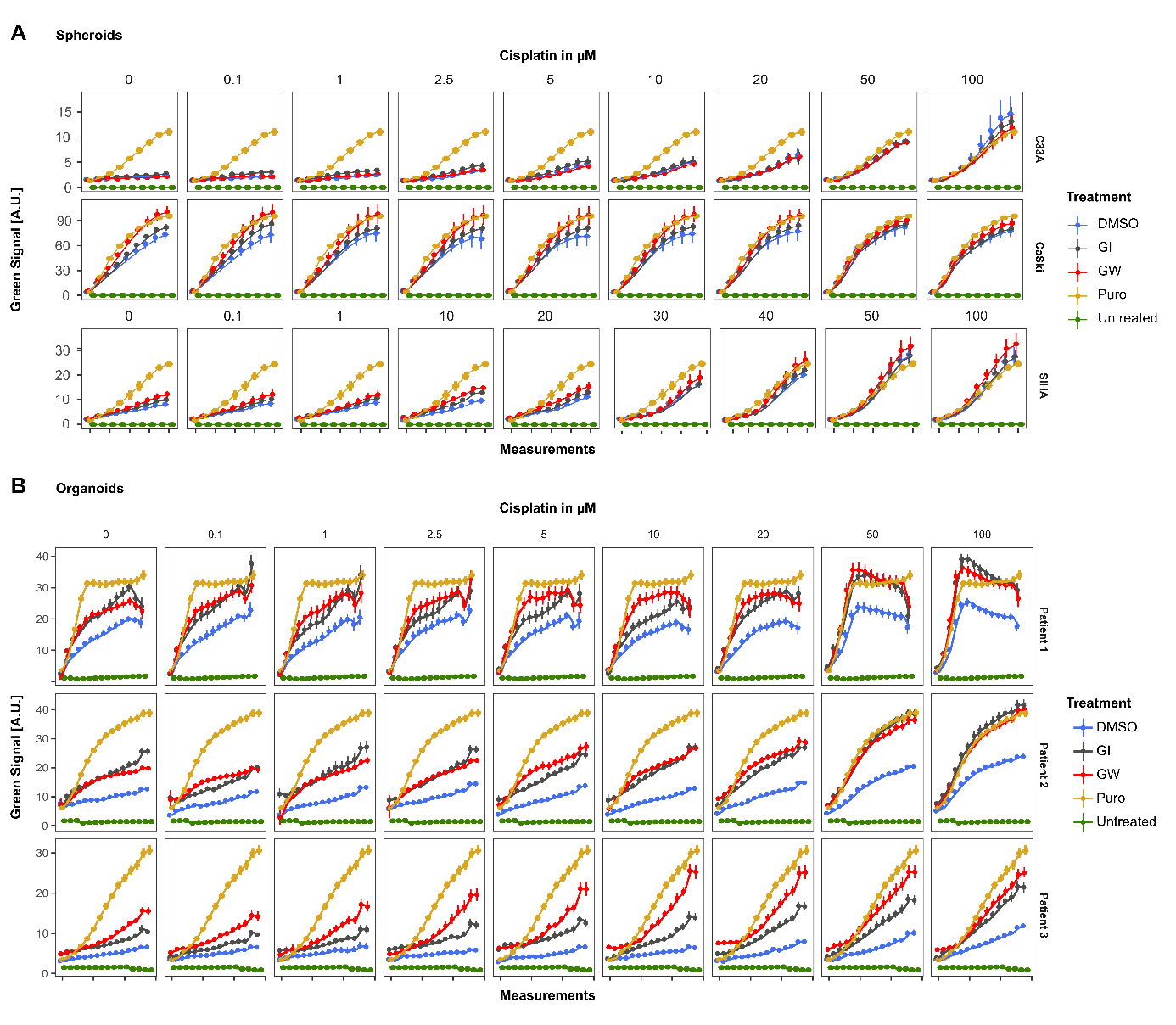


Supplementary Figure 4: Cytotoxicity measured by live cell imaging with CellTox Green over time. Varying Cisplatin concentrations with/without 3µM ADAM10 inhibitor GI254023X or ADAM10/17 inhibitor GW280264X are displayed. Data shows mean (± 95% CI) from ≥ 3 independent experiments per patient or cell line. 2 µg/ml of Puromycin was included as a positive control for cytotoxicity. Untreated controls indicate unstained and untreated organoids.

# Supplementary Tables

Supplementary Table 1: Patient characteristics of the organoid lines.

| **ID** | **Age at surgery** | **Condition** | **HPV status** |
| --- | --- | --- | --- |
| Patient 1 | 35 | Squamous cell carcinoma | HPV16+ |
| Patient 2 | 54 | Squamous cell carcinoma | HPV16+ |
| Patient 3 | 61 | Squamous cell carcinoma | HPV18+ |

Supplementary Table 2: Antibodies, dyes and dilutions.

| **Target** | **Host** | **Vendor** | **Application** | **Dilution** |
| --- | --- | --- | --- | --- |
| **Primary antibodies** | | | | |
| KRT5-A488 | Rabbit | Abcam, ab193894, RRID:AB_2893023 | IF | 1:500 |
| KI67 | Rabbit | Abcam, ab16667, RRID:AB_302459 | WB | 1:1,000 |
| P53 | Mouse | Santa Cruz (DO-1), sc-126, RRID:AB_628082 | WB | 1:1,000 |
| ADAM17 | Goat | R&D Systems, AF9301, RRID:AB_10891879 | WB | 1:1,000 |
| ACTB | Mouse | Sigma, A5441, RRID:AB_476744 | WB | 1:10,000 |
| **Secondary antibodies** | | | | |
| Anti-Goat HRP | Donkey | Abcam, ab97110, RRID:AB_10679463 | WB | 1:3,000 |
| Anti-Rabbit HRP | Goat | Cell Signalling, 7074S, RRID:AB_2099233 | WB | 1:3,000 |
| Anti-Mouse HRP | Horse | Cell Signalling, 7076S, RRID:AB_330924 | WB | 1:3,000 |
| **Dyes** | | | | |
| Hoechst33342 | - | Thermo Fisher, H1399 | IF/Live cell imaging | 5 µg/ml |
| Propidium iodide | - | Miltenyi Biotec, 130-093-233 | Live cell imaging | 0.2 µg/ml |
| Calcein Green | - | Invitrogen, C34852 | Live cell imaging | 1 ng/ml |
| CellTox Green | - | Promega, G8741 | Live cell imaging | 1:2,000 |
